# Advanced Technologies in Extracellular Vesicle Biosensing: Platforms, Standardization, and Clinical Translation

**DOI:** 10.3390/molecules31020227

**Published:** 2026-01-09

**Authors:** Seong-Jun Choi, Jaewon Choi, Jin Kim, Si-Hoon Kim, Hyung-Geun Cho, Min-Yeong Lim, Sehyun Chae, Kwang Suk Lim, Suk-Jin Ha, Hyun-Ouk Kim

**Affiliations:** 1 Division of Chemical Engineering and Bioengineering, College of Art, Culture and Engineering, Kangwon National University, Chuncheon 24341, Republic of Korea; tdjwns3@gmail.com (S.-J.C.); choijw8839@kangwon.ac.kr (J.C.); kimsihoon38@gmail.com (S.-H.K.); yes13149@gmail.com (H.-G.C.); minyeongim205@gmail.com (M.-Y.L.); shchae@kangwon.ac.kr (S.C.); kslim@kangwon.ac.kr (K.S.L.); 2 Department of Smart Health Science and Technology, College of Engineering, Kangwon National University, Chuncheon 24341, Republic of Korea; happyrlawls@kangwon.ac.kr; 3 Institute of Fermentation of Brewing, Kangwon National University, Chuncheon 24341, Republic of Korea; 4 Institute of Industrial Technology, Kangwon National University, Chuncheon 24341, Republic of Korea

**Keywords:** extracellular vesicle, extracellular vesicle-based biosensing, point-of-care diagnosis

## Abstract

Recently, extracellular vesicles (EVs) have emerged as pivotal mediators of intercellular communication that reflect physiological homeostasis and pathological alterations. By encapsulating diverse biomolecules, including proteins, nucleic acids, and lipids, EVs mirror the molecular signatures of their parent cells, thereby positioning EV-based biosensing as a transformative platform for noninvasive diagnostics, prognostic prediction, and therapeutic monitoring. This review provides a comprehensive overview of the current state and clinical translation of EV biosensing technologies. Herein, we have discussed ongoing efforts toward standardization and analytical validation (e.g., MISEV2023 and EV-TRACK) and evaluated advances in sensing modalities such as surface plasmon resonance (SPR), electrochemical, fluorescence, and magnetic detection systems, which have significantly improved analytical performance in terms of sensitivity and specificity. Furthermore, we highlight recent developments in multiplexed and multiomics integration at the single-EV level and the application of machine learning to enhance diagnostic accuracy and interpret biological heterogeneity. The clinical relevance of EV biosensing has been explored across multiple disease domains, including oncology, neurology, and cardiometabolic and infectious diseases, with an emphasis on translational progress toward standardized, regulatory-compliant, and scalable platforms. Finally, this review identifies key challenges in manufacturing scale-up, quality control, and point-of-care deployment and proposes a unified framework to accelerate the adoption of EV biosensing as a cornerstone of next-generation precision diagnostics and personalized medicine.

## 1. Introduction

### 1.1. Rationale and Clinical Relevance of EV Biosensing

EVs are key mediators of intercellular communication that carry various biomolecules such as proteins, nucleic acids, and lipids, reflecting the physiological or pathological state of their originating cell [1,2]. This unique property enables them to serve as essential biomarkers for disease diagnosis, prognostic prediction, and therapeutic monitoring [3,4]. EV-based biosensing technologies have emerged as promising noninvasive diagnostic tools that can overcome the limitations of conventional tissue biopsy, demonstrating potential applications in oncology, neurology, infectious diseases, and metabolic disorders [3,5]. For instance, PD-L1 proteins expressed on tumor-derived EVs have been proposed as biomarkers for predicting immunotherapy responses [6,7], while the ability of EVs to cross the blood–brain barrier has attracted interest for early diagnosis and targeted drug delivery in neurological diseases [2,8]. Moreover, a circulating EV-based liquid biopsy minimizes temporal and physical burdens, making it an innovative diagnostic approach that aligns with the goals of precision medicine [3,4]. By quantitatively analyzing physiological and pathological states, EV biosensing is evolving into a core technology for personalized disease management and is anticipated to drive a paradigm shift in clinical diagnostics and therapeutic monitoring [4,9].

### 1.2. Definitions, Nomenclature, and EV Classes

EVs are nanoscale membrane-bound vesicles released from cells and are broadly categorized into exosomes, microvesicles, and apoptotic bodies according to their size and biogenesis [1,2]. Exosomes are 30–150 nm vesicles derived from the fusion of multivesicular bodies (MVBs) with the plasma membrane, whereas microvesicles (100–1000 nm) are formed through direct outward budding of the plasma membrane [1,2]. In contrast, apoptotic bodies are larger vesicles (500–2000 nm) generated during programmed cell death that may contain fragmented DNA or organelles [1]. Historically, the terminology used to describe EVs has been inconsistent; however, the International Society for Extracellular Vesicles (ISEV) has established “minimal information for studies of Extracellular Vesicles (MISEV)” to promote standardization [9]. The guidelines recommend clear specifications of EV origin, size, surface markers (e.g., CD9, CD63, and CD81), isolation methods, and transparent reporting of experimental conditions to ensure reproducibility and reliability [9,10]. Such classification and standardization provide a foundation for scientific rigor and clinical applicability in EV research, particularly in biosensing, where understanding the physicochemical and biological diversity of EVs enables the development of highly specific and sensitive analytical technologies [3,9,10].

### 1.3. Summary of This Reviewr

This review comprehensively covers the biological basis, biosensing technologies, and clinical applications of EVs, with the aim of delineating the current state and prospects of EV biosensing [1,9]. It begins by outlining the fundamental biological properties and classification systems of EVs, followed by an in-depth discussion of the major technological platforms for EV isolation, purification, and detection, as well as their respective advantages and limitations [3,10]. This review examines recent advances in EV-based biosensing technologies, including electrochemical, optical, and nanophysical approaches, and highlights their roles in enhancing diagnostic performance [3,5]. Furthermore, it addresses the clinical translation of EV biosensing across various fields, such as oncology, neurology, cardiometabolic diseases, and infectious diseases, emphasizing the significance of standardization frameworks such as MISEV and EV-TRACK, as well as regulatory considerations for diagnostic implementation [4,9]. Finally, the paper explores future directions encompassing large-scale manufacturing, automation, point-of-care (POC) integration, AI-assisted quality control, and globally standardized distributed production networks [3,9]. By integrating these multidisciplinary insights, this review aims to provide a comprehensive perspective on the establishment of EV-based biosensing as a pivotal platform for next-generation precision diagnostics and personalized medicine [4,9].

## 2. Biology of Extracellular Vesicles and Biomarker Space

### 2.1. Biogenesis, Heterogeneity, and Cargo Composition

**Figure 1:**
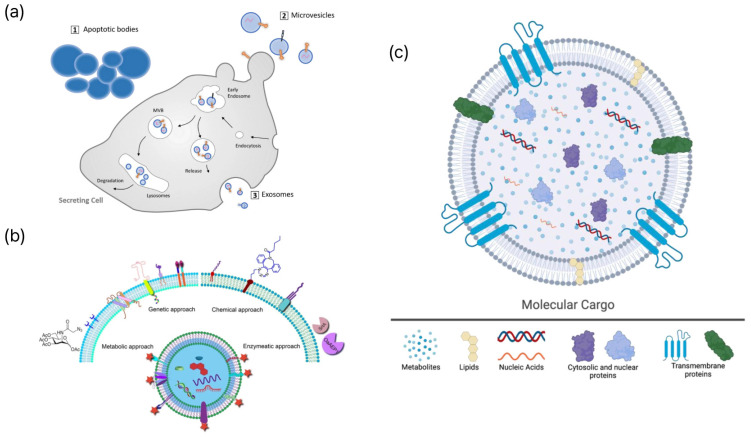
Schematic representations of EV biogenesis, engineering strategies, and molecular cargo. (**a**) Biogenesis pathways of major extracellular vesicle subtypes, including apoptotic bodies, microvesicles, and exosomes. Adapted from [22]. (**b**) Representative engineering strategies for EV surface or cargo modification, encompassing genetic, chemical, metabolic, and enzymatic approaches. Adapted from [23]. (**c**) Molecular constituents of extracellular vesicles, including metabolites, lipids, nucleic acids, cytosolic proteins, and transmembrane proteins. Reproduced from [24].

Enhancing EV-based tests and treatments requires a comprehensive understanding of EV biogenesis, heterogeneity, and molecular composition. Cells release EVs and membrane-bound entities through intricate biological processes. They are typically categorized into three groups based on their formation: exosomes, ectosomes, and apoptotic bodies (Figure 1a). Exosomes, which are typically less than 150 nm in diameter, are derived from MVBs created by the inward budding of the endosomal membrane [11]. Exosomes are released into the extracellular environment through the fusion of MVBs with the plasma membrane [12]. Two mechanisms are involved in their formation: one relies on the Endosomal Sorting Complex Required for Transport (ESCRT), and the other operates independently. The ESCRT-dependent mechanism involves the use of ESCRT-0, ESCRT-I, ESCRT-II, and ESCRT-III complexes, whereas the ESCRT-independent pathway is characterized by tetraspanin-enriched microdomains (CD9, CD63, and CD81) and ceramide-induced membrane curvature [10]. Ectosomes, also referred to as microvesicles, are EVs ranging from 100 to 1000 nm in size that are formed through the budding of the plasma membrane [13]. Lipid content, cytoskeletal dynamics, and calcium signaling contribute to size determination. Apoptotic bodies, released following programmed cell death, range in size from 50 nm to 3 μm and may contain fragmented DNA and cellular organelles (Table 1). EVs have various sizes, materials, and applications. Multiple biogenetic processes can occur simultaneously within a single cell. These pathways include ESCRT-dependent, ESCRT-independent, and calcium-regulated ectosomal mechanisms [14]. External stimuli may influence these pathways, thereby altering EV properties. EV payloads comprise proteins, lipids, and nucleic acids that mirror the physiological state of their source cells and affect the behavior of the recipient cells (Figure 1c). Rab GTPases, SNAREs, and flotillins are commonly identified in EVs. Glycosylation influences protein stability and the binding affinity for other molecules. EVs encompass single-stranded DNA (ssDNA), double-stranded DNA (dsDNA), mitochondrial DNA (mtDNA), messenger RNAs (mRNA), long noncoding RNAs (lncRNAs), and microRNAs (miRNAs), such as miR-222 and miR-143, which may function as potential biomarkers [15]. The lipid bilayer, which is composed of sphingolipids and cholesterol, protects the intracellular components and enhances the specificity for certain target cells. EVs play a crucial role in intercellular communication and precision medicine because of their diverse chemical and functional characteristics. The inherent heterogeneity of EVs, arising from diverse cellular origins and biogenetic pathways, presents a significant challenge for biomarker specificity. Bulk analysis often provides an ensemble average, which may obscure rare but clinically significant subpopulations carrying high-specificity disease markers. To overcome this limitation, single-EV analysis has emerged as a transformative approach. For instance, digital assays and proximity-dependent barcoding have demonstrated that only a small fraction (often <1%) of circulating EVs carry tumor-specific markers such as EGFRvIII or PD-L1 [16]. These multiplexed single-vesicle platforms significantly improve the area under the curve (AUC) for early cancer detection by effectively filtering out the noise from non-specific host-derived EVs [17]. Such granular understanding of EV heterogeneity is essential for improving the signal-to-noise ratio in next-generation biosensing.

**Table 1:** Features of extracellular vesicles.

EV Subtypes	Origin	Size (nm)	Biomarkers	Synonym	Ref.
Exosomes	Multivesicle body	50–150	CD9, CD63, Tsg101, CD81, ALIX, HSP70	Small EV/sEV	[1,10,18]
Microvesicles	Plasma membrane	10–1000	Integrins, Selectins, CD40, tissue factor	Ectosome, shed vesicle	[18,19]
Apoptotic bodies	Plasma membrane	100–5000	Annexin V, C3b, thrombospondin, Annexin A1, histone coagulation factor	Apoptotic vesicle	[18,20,21]

### 2.2. Molecular Targets: Surface Proteins, Nucleic Acids, Lipids, and Glycans

EVs are important intermediaries in liquid biopsies and reflect the physiological and pathological states of their source cells [1]. Developing high-precision diagnostic and monitoring systems that employ EVs requires an in-depth understanding of the molecular targets that can distinguish between heterogeneous EV subpopulations. Surface proteins, nucleic acids, lipids, and glycans are critical molecular targets within EV components that affect the sensitivity and specificity of biosensing technologies (Figure 1b). Surface proteins are closely linked to EV biogenesis and cellular origin and are widely used for the identification of EVs [10]. Proteins associated with ESCRT, such as ALIX and TSG101, are common exosome markers. Tetraspanins, including CD9, CD63, and CD81, are integral to ESCRT-independent pathways and are consistently enriched in EV membranes. Surface proteins specific to cell types, including receptors or ligands present on tumor-derived EVs, facilitate selective detection through antibody-based sensors, such as SPR or ELISA microarrays, thus contributing to disease monitoring. EV nucleic acids are protected by lipid membranes that inhibit enzymatic degradation and maintain the stability of genetic information reflective of the parent cell [25]. miRNAs are extensively studied biomarkers that display disease-specific expression patterns, enabling their sensitive detection by qPCR or digital PCR [26]. In addition, lncRNAs, mRNAs, and DNA components, including dsDNA and mtDNA, found in EVs demonstrate diagnostic potential as indicators of genomic instability. The EV lipid bilayer, rich in cholesterol, sphingolipids, and ceramides, is essential for vesicular stability and formation. Externalized phosphatidylserine (PS) on apoptotic bodies serves as a detection target using Annexin V probes [27]. Furthermore, glycans linked to EV proteins and lipids play a role in complex molecular signatures. Tumor-derived EVs often exhibit unusual glycosylation patterns, including altered sialylation and fucosylation, which are associated with cancer progression. Lectin-based sensors employ specific interactions between lectins and glycans to distinguish EV subtypes. The various biomolecular constituents of EVs establish a unique molecular signature that reflects their cellular sources, thus facilitating multiplex biosensing and sophisticated liquid biopsy methodologies [13]. While EV engineering strategies (Figure 1b) are primarily discussed in this review for enhancing biosensing performance, these modifications are increasingly intersecting with therapeutic applications. Genetic modification of parent cells to overexpress targeting ligands, such as RVG peptides or nanobodies, enables EVs to cross biological barriers for site-specific drug delivery [8]. Furthermore, chemical functionalization via click chemistry allows for the loading of therapeutic cargoes, including siRNA or chemotherapeutic agents, into the EV lumen [28]. This synergy between engineering for sensing and therapy underscores the versatile role of EVs in next-generation precision medicine, where the same modification can serve both diagnostic and therapeutic purposes.

### 2.3. Pre-Analytical Variables and Sample Matrices

EVs serve as crucial elements in liquid biopsies, transporting molecular data that reflect the physiological and pathological conditions of their source cells [29]. This distinct property has enabled the development of EV-based biosensing technologies. The accuracy and reproducibility of EV analysis are significantly influenced by pre-analytical variables ranging from sample collection to processing, as well as the complexity of biological matrices [30]. Uncontrolled handling may distort EV profiles and compromise diagnostic reliability. Standardized pre-analytical workflows and matrix-optimized protocols are crucial for ensuring clinical applicability [30]. The selection of anticoagulants for blood-derived samples is essential. EDTA chelates Ca^2+^, which may destabilize EV membranes and induce artificial EV release, whereas citrate causes a lesser degree of disruption. Delays and temperature variations before processing can activate platelets and leukocytes, leading to the generation of artificial EVs. Consequently, plasma or serum must be separated promptly utilizing standardized centrifugation protocols (≤2000× *g* for cells, 10,000–20,000× *g* for platelets). The isolation method significantly affects the yield and purity of EVs [30]. Ultracentrifugation (UC) is commonly employed; however, it may compromise EVs and co-sediment lipoproteins, thereby diminishing their purity [31]. Size-exclusion chromatography (SEC) reduces contamination, but requires longer processing times, whereas immunoaffinity capture using CD9, CD63, or CD81 antibodies provides specificity, albeit potentially at the cost of yield [10]. Integration of various isolation methods can reduce this bias. Freeze–thaw cycles negatively affect EV stability by inducing ice crystal formation and osmotic stress, resulting in membrane disruption and RNA degradation. To mitigate this issue, samples must be aliquoted, stored at −80 °C or in liquid nitrogen, and may be stabilized with trehalose. Matrix complexity presents significant analytical challenges. Plasma and serum contain lipoproteins with sizes and densities comparable to those of EVs, resulting in background noise in biosensing assays [32]. Therefore, further purification was required. Plasma more accurately represents physiological EV profiles, whereas serum may exhibit artificially increased EV counts owing to platelet activation. Other fluids such as urine, cerebrospinal fluid, and saliva exhibit low EV abundance or pH variability, necessitating customized processing methods. Controlling pre-analytical parameters, such as anticoagulant type, processing delay, freeze–thaw stress, and matrix interference, is essential for maintaining the integrity of EVs. Future advancements will rely on the automation and miniaturization of these processes to ensure consistent high-purity recovery of EVs from various biological samples.

## 3. Preprocessing and Enrichment Strategies

### 3.1. Conventional Isolation: Ultracentrifugation, Size-Exclusion, Precipitation

**Figure 2:**
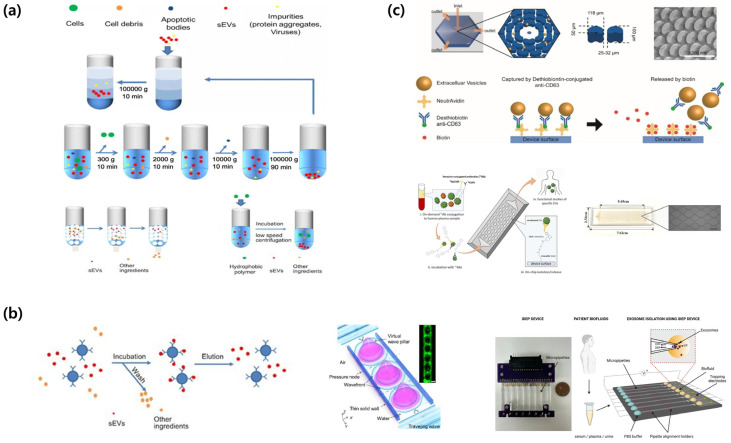
Schematic overview of small extracellular vesicle (sEV) isolation and purification methods. (**a**) Conventional isolation methods for extracellular vesicles include isopycnic density gradient centrifugation, differential ultracentrifugation, size-exclusion chromatography, and polymer-based precipitation for sEV collection. Adapted from [41]. (**b**) Affinity- and property-based enrichment strategies for extracellular vesicle isolation encompass immunoaffinity capture technology, acoustofluidic nanosorters utilizing wave-pillar resonance mechanisms, and insulator-based dielectrophoretic (iDEP) devices. Adapted from [41,42,43]. (**c**) On-chip and in situ capture workflows: Schematic illustration of OncoBean Chip and EV isolation, working principle of exosome isolation using EVOD chips and photographic image of fabricated EVOD devices. Reproduced with permission from [44,45].

UC is the principal method for isolating EVs, and is classified into differential and density-gradient centrifugation techniques [33,34]. Differential centrifugation involves the initial separation of cell debris at 2000× *g*, followed by UC at 100,000–200,000× *g* for 1–2 h to isolate EVs as a pellet (Figure 2a) [33,34]. Density-gradient centrifugation utilizes sucrose or iodixanol gradients (1.10–1.19 g/mL) for the isolation of EVs with high purity (Figure 2a) [33]. This method offers several advantages, including the capacity to manage large sample volumes (tens of milliliters), maintain the structural and functional integrity of EVs, and operate exclusively on physical properties, eliminating the requirement for chemical reagents [33,35]. Several drawbacks exist, including extended processing times (>8 h), low recovery yields (5–25%), contamination by proteins and lipids, substantial costs associated with UC equipment and consumables, and inadequate reproducibility among laboratories [33,36]. Moreover, high-speed centrifugation can lead to aggregation or deformation of EVs, which may impact subsequent functional analyses [33,37,38]. SEC differentiates EVs from proteins by utilizing the particle size and employing columns filled with porous resins (Figure 2a) [33,35]. EVs that cannot penetrate pores elute initially, whereas smaller proteins traverse the resin and elute subsequently [33,35]. Commercial qEV columns enable separation within 30–60 min, resulting in enhanced purity and recovery yields of EVs compared with UC methods [35]. SEC offers several advantages, including effective elimination of protein contaminants, maintenance of EV structural integrity and bioactivity, and operation through gravity, which eliminates the requirement for specialized or costly equipment [35,39]. The method is constrained by its sample loading capacity, which ranges from approximately 200 to 500 μL per column [35]. This limitation necessitates multiple runs for large-volume samples and an additional concentration step to mitigate EV dilution [35]. The complete separation of lipoproteins of comparable sizes remains a considerable challenge [35,36]. Methods for isolating EVs through precipitation involve reducing the surrounding hydration layer using high-molecular-weight polymers, such as polyethylene glycol (PEG) (Figure 2a) [33,39,40]. Commercially available kits, such as ExoQuick and Total Exosome Isolation (TEI), facilitate the recovery of EVs via centrifugation following incubation at 4 °C for several hours to overnight [35,39,40]. This method is simple, has a quick turnaround time of 2–12 h, and is appropriate for processing large sample volumes of at least 1 mL without requiring costly equipment [33,39,40]. However, this method is constrained by its low purity, interference from residual reagents in subsequent analyses, coprecipitation of proteins and lipid particles, and diminished specificity for EVs [33,35,40].

### 3.2. Affinity- and Property-Based Enrichment: Immunocapture, Acoustics, Dielectrophoresis

Immunocapture-based isolation utilizes antibodies that selectively bind to surface markers, including tetraspanins CD9, CD63, and CD81, as well as cell type-specific proteins such as EpCAM and PSMA (Figure 2b) [33,46]. Antibodies are used on various substrates, such as plates, magnetic beads, and microfluidic chips [33]. This method enables the precise isolation of EVs from designated cell types, yielding high specificity and purity [33]. This method is effective for complex biological materials requiring minimal pretreatment [47]. Further benefits include the capacity to simultaneously target multiple markers and operation of automated chip-based systems [33,47]. This technique delineates distinct EV sub-populations of extracellular vesicles, thereby reducing the overall representation of the EV category [33,46]. Antibodies incur significant costs, and there is the potential for structural damage during the release of EVs [33]. Acoustic-based separation employs surface acoustic waves (SAWs) or bulk acoustic waves (BAWs) to generate acoustic radiation pressure on the EVs, thereby facilitating their separation without direct contact [46,48,49]. Multiple methods have been proposed including floating acoustic trapping (FLOAT), acoustic trapping, and acoustophoresis [46,48]. An acoustofluidic nanosorter by wave-pillar resonance (ANSWER) technology employs virtual acoustic wave pillars for sorting nanoparticles with diameters less than 50 nm (Figure 2b) [48]. This method functions in under 10 min and attains recovery rates exceeding 90%, maintaining the structural and biological integrity of EVs without requiring tagging [48,49]. The approach encounters multiple challenges, such as elevated initial equipment costs, restricted throughput within the μL–mL range, and the risk of sample degradation at high sonic power levels [48,49]. Dielectrophoresis (DEP) facilitates the separation of EVs by applying force on polarized particles within a non-uniform electric field [36,46,50]. Insulator-based dielectrophoresis (iDEP) employs insulating microstructures in a microchannel to create localized electric field gradients, thereby facilitating the movement of EVs (Figure 2b) [46,50]. EVs experience dielectrophoretic forces that can be either positive or negative depending on their size, surface charge, and dielectric properties, thereby facilitating selective separation [36,46,50]. DEP does not require labeling, operates within minutes, and can be integrated into microfluidic chips, thereby facilitating the development of more compact and automated systems [46,49,50]. The magnitude and frequency of the electric field can be adjusted to dynamically alter the separation parameters [36,46]. This facilitates the classification of particles according to their size and dielectric characteristics [36,46,50]. The increased ionic strength of biological samples diminishes the efficacy of the electric field, which requires either sample dilution or buffer modification [36,46,50]. The high-voltage operation in electric vehicles may result in overheating and bubble formation, which can potentially cause damage [36,46]. Compared to UC, this process exhibits lower throughput, an increased risk of channel obstruction, and inadequate consistency [36,46,51].

### 3.3. On-Chip and In Situ Capture Workflows

On-chip and in situ methods integrate isolation, concentration, and detection within a unified microfluidic platform [33,46,47]. The ExoChip employs channels coated with anti-CD63 antibodies to capture and quantify serum-derived EVs [33,46]. The OncoBean chip improves EV collection efficiency by incorporating radial flow and bean-shaped microposts, thereby augmenting the available surface area (Figure 2c) [46,49,50]. Viscoelastic microfluidic technologies facilitate label-free separation of EVs smaller than 200 nm directly from blood samples [46,50]. The EVs On Demand (EVOD) chip integrates click chemistry with dithiothreitol (DTT)-mediated release, facilitating efficient capture and release of EVs (Figure 2c) [46,47,50]. Microfluidic platforms require minimal sample preparation, facilitate rapid analysis within 5–30 min, and utilize only microliter-scale sample volumes [33,46,50]. Their automation and portability render them suitable for POC diagnosis [46,47,49]. These methods enhance sensitivity and specificity while minimizing EV loss during sample preparation [46,47,51]. The feasibility of real-time analysis and multiplex detection of multiple biomarkers has been demonstrated. The throughput of these platforms is low, varying from several microliters to several hundred microliters. Challenges encompass the complexity of chip fabrication, issues related to channel clogging, lack of standardization and validation, and elevated costs associated with disposable chips [33,47,51]. Future initiatives should focus on optimizing manufacturing processes, developing high-throughput applications, and conducting validation studies to guarantee standardization and clinical implementation [33,38,47].

## 4. Assay Design and Sensing Mechanisms

### 4.1. Recognition Elements: Antibodies, Aptamers, Peptides, Membrane-Mimetic Ligands

In the analysis of single EVs, recognition elements are essential for accurate isolation and selective capture of target vesicles. Antibody-based co-targeting strategies that simultaneously target CD9, CD63, and CD81 effectively mitigate vesicle heterogeneity and serum interference, resulting in significantly enhanced detection sensitivity compared with single-antibody methods [9]. A dual monoclonal antibody that targets CD81 and CD63 showed enhanced positivity rates for small EVs (under 100 nm), effectively mitigating size-dependent capture bias [9]. An antibody-aptamer combination (AAC) merges the specificity of antibodies with the chemical stability of aptamers, thereby enabling selective extraction and quantification of EV subpopulations in complex matrices [52,53]. Affinity has been improved through the systematic development of ligands using exponential enrichment (SELEX), cell-SELEX, and capture-SELEX techniques [52,53]. The PD-L1 aptamer-based traceless isolation technique enables the release of label-free PD-L1^+^ small EVs through competitive complementary oligonucleotide treatment, allowing for the profiling of adhesion and immune-regulatory molecules [53]. Peptide-based recognition utilizes the coil-to-α-helix transition of amphipathic helical peptides to engage with high-curvature membranes, whereas unstapled curvature-sensing peptides are capable of crossing bacterial EV polysaccharide barriers for detection purposes [54,55]. A net charge-invertible curvature-sensing peptide (NIC) enables a pH-dependent capture-and-release system to isolate EV-CaRiS, allowing repeatable single-particle separation via total internal reflection fluorescence (TIRF) microscopy [56]. Membrane-mimetic ligands, such as lipid-polymer conjugates and peptide-lipid hybrids, emulate membrane interfaces, demonstrating dissociation constants (Kd) approximately 10^−8^ M and detection limits (LOD) as low as 10^3^ particles/mL [54,57]. The theranostic capabilities of tumor-derived EVs were improved by pH-triggered drug release mechanisms [57].

### 4.2. Transduction and Amplification: Electrochemical, Optical, Mechanical; Enzymatic/DNA/CRISPR

To achieve single-vesicle sensitivity, extracellular vesicle (EV) binding events are converted into measurable signals through integrated physical, chemical, and nucleic acid-based strategies. Electrochemical transduction platforms are highly effective for point-of-care applications due to their high sensitivity. For instance, sensors based on electrochemical quartz crystal microbalance with dissipation integrate a two-dimensional gold nanoarray to achieve a fourfold increase in sensitivity by simultaneously monitoring mass, viscoelasticity, and electrochemical signals [58]. Other electrochemical modalities, including square wave voltammetry and differential pulse voltammetry, have been utilized with paper-based electrodes to achieve detection limits below 0.7 × 10^3^ EVs/mL and broad dynamic ranges for clinical diagnostics [59,60]. Optical transduction techniques provide high-resolution insights into vesicle heterogeneity and molecular signatures. Total internal reflection fluorescence microscopy enables repeatable single-particle imaging, while super-resolution modalities—including stimulated emission depletion, stochastic optical reconstruction microscopy, and super-resolution radial fluctuation nanoscopy—allow for the precise analysis of EV size, frequency, and biomarkers [61]. Furthermore, surface-enhanced Raman spectroscopy leverages localized surface plasmon resonance to amplify Raman signals by 10^10^–10^11^-fold for label-free multiplex detection [18,19,20,21]. This approach has demonstrated a detection accuracy of 97.4% for ovarian cancer–derived small EVs, achieving a limit of detection of 1.5 × 10^5^ particles/μL particles/μL [62]. Mechanical and molecular amplification strategies further enhance analytical performance for low-abundance targets. Three-dimensional atomic force microscopy is employed to quantify EV elastic moduli (50–350 MPa) and elucidate the properties of the extracellular matrix during metastasis [61]. At the molecular level, signal amplification is increasingly achieved through isothermal DNA-based circuits. Strategies such as the hybridization chain reaction can be integrated with CRISPR-associated protein 12a trans-cleavage to achieve a detection limit of 10^2^ particles/μL. Similarly, exponential amplification reactions utilize the CRISPR-Cas9 system and single-guide RNA to detect exosomal miRNA-21 at concentrations as low as 3 × 10^3^ particles/mL [52]. These enzyme-free assembly processes, including catalytic hairpin assembly, ensure high efficiency and biocompatibility in complex biological matrices [52].

### 4.3. Surface Chemistry, Antifouling, and Matrix-Effect Mitigation

Surface modification and antifouling techniques are crucial for reducing nonspecific adsorption and alleviating matrix effects in complex biological fluids. A micrometer-thick porous nanocomposite coating featuring albumin crosslinking and interconnected pores demonstrated sustained antifouling performance for over one month, resulting in an enhancement of detection sensitivity by approximately 3.8–17 times [63]. A self-assembled monolayer (SAM) of Si-MEG-OH on gold electrodes demonstrated antifouling properties similar to those of polymer brush coatings [63]. Self-assembled peptide films on platinum nanoparticles enhance the signal stability and reduce the limit of detection [54,55]. Zwitterionic polymers, PEG, and antifouling peptides inhibit nonspecific protein adsorption through the formation of hydration shells and the generation of steric repulsion [54,63]. The development of printed antifouling electrodes enables large-scale and cost-effective production of sensors [59,60]. Engineered hydrogels that replicate the mechanical softness of native tissues can enhance the secretion of EVs from mesenchymal stem cells (MSCs) by a factor of up to 10. This resulted in an improved vesicle output and uniformity [63]. Preclinical studies demonstrated that matrix-bound nanovesicles (MBVs) exhibit significant anti-inflammatory properties [63]. Biosensors utilizing molecularly imprinted polymers (MIPs) integrate internal reference signals to reduce matrix interference during the detection of SARS-CoV-2 spike proteins [64,65]. Self-powered enzymatic biofuel cell sensors operate independently of the external power sources. This ensures baseline stability, which is crucial for reliable POC diagnostics [57] (Table 2).

## 5. Platform Technologies for EV Detection

### 5.1. Electrochemical and Electrical Biosensors

Electrochemical biosensors are useful for identifying EVs because they can accomplish this in real time without labels and are very sensitive and cheap [74,75]. The conversion of EV–electrode interactions into measurable electrical signals enables the detection of limits in the attomolar range through advanced amplification techniques such as AuNP modification (4.31 aM for miRNA-21-5p), evaporation-enhanced redox cycling (E2RC, 1.2 × 10^3^ particles/mL), and DNA tetrahedron-assisted CHA (25 aM) [74,76,77,78]. These platforms can be utilized in several ways. For instance, immunomagnetic bead-based systems measure EVs specific to a disease at clinically relevant concentrations (>10^8^ particles/mL) [75,79]. Perovskite-modified electrodes focus on cardiac biomarkers for myocardial infarction, and AlGaN/GaN HEMT biosensors enable direct detection under physiological conditions without significant preprocessing [74,75]. Machine learning has enabled the diagnosis of stomach cancer with an accuracy > 88% [74]. Wearable electrochemical biosensors are also improving, making it feasible to keep an eye on your health at all times and in a way that is unique to you [80]. POC applications are promising because electrochemical platforms can be made smaller, have quick turnaround times (<30 min), and function with multiplexed detection [74,75]. However, clinical implementation faces several challenges such as matrix effects and biofouling, which complicate the analysis of sophisticated biofluids. The reproducibility of these findings is hindered by the absence of standardized reference materials for electric vehicles, which are produced using various methods. Signal drift presents challenges for applications that require continuous monitoring to maintain stability over time. Detection limits are typically superior in spiked samples or model systems than in real clinical matrices, where lipoproteins, protein aggregates, and cellular debris may lead to false positives [74,75]. To rectify this deficiency, it is crucial to create standardized surface chemistries that exhibit strong antifouling properties, implement scalable manufacturing processes, and perform comprehensive multicenter validation involving diverse patient cohorts [74,75].

### 5.2. Optical and Spectroscopic Biosensors (SPR/LSPR, SERS, Interferometry, Fluorescence)

**Figure 3:**
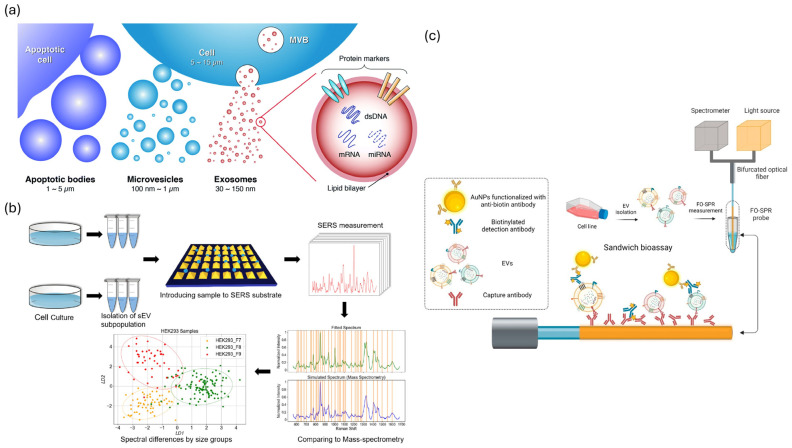
Optical and spectroscopic biosensing strategies for extracellular vesicle (EV) detection. (**a**) Types and molecular contents of EVs, including apoptotic bodies, microvesicles, and exosomes. Reproduced with permission from [86]. (**b**) SERS−based workflow for EV profiling from cell culture to spectral classification. Adapted from [87]. (**c**) FO−SPR sandwich bioassay using AuNPs for label-free, real-time EV detection. Adapted from [83].

Optical biosensors utilize plasmonic and photonic phenomena for label-free, non-invasive detection of EVs, achieving single-vesicle resolution and enabling real-time kinetic profiling [81,82]. SPR and LSPR technologies have evolved into platforms of clinical significance [81,82,83,84,85]. Fiber-optic SPR facilitates the direct analysis of plasma EVs linked to breast and ovarian cancers [83]. Compact SPR instruments have lower cost barriers while preserving picomolar sensitivity [84]. LSPR biosensors offer portable and cost-effective alternatives that demonstrate a significant correlation with gold-standard ELISA results (Figure 3a) [82,85]. The incorporation of advanced nanomaterials such as gold nanoparticles, graphene, and carbon nanotubes has markedly improved detection limits, stability, and specificity [81,82,85]. Microfluidic coupling facilitates the concurrent detection of inflammation biomarkers (CRP, IL-6, TNF-α), with clinical validation indicating 95% specificity and 73% sensitivity in patient samples [82,84]. SERS platforms facilitate molecular fingerprinting and attain femtomolar sensitivity by enhancing electromagnetic hot spots [81,82,85].” Recent advancements in label-free SERS systems have effectively characterized EV heterogeneity by correlating particle size with enhancer size (Figure 3b) [81,85]. illustrates the multiparametric platforms that integrate fluorescence, SPR, and Raman spectroscopy, enabling a thorough analysis of EV subpopulations [81,82]. Recent advancements in plasmonic waveguide design have led to a 20-fold enhancement in the signal detection of ultrasmall biomolecules [81]. Figure 3b illustrates the advancements in clinical adoption. SPR biosensors assess therapeutic responses in patients with ovarian cancer, identify inflammatory biomarkers at the point-of-care, and facilitate rapid screening for sepsis and autoimmune disorders [82,83]. Figure 3c illustrates that commercial LSPR platforms exhibit reproducibility (CV 3.5–9.3%) and recovery (101–105%) comparable to those of ELISA, while also offering faster turnaround times and requiring smaller sample volumes [82,85]. Translational challenges continue to exist, and morphological variations in nanostructure synthesis lead to interbatch inconsistencies that undermine reproducibility. Fluctuations in the bulk refractive index and non-specific protein adsorption in complex matrices impede the detection of EV-specific signals. Quantification is challenging because of the lack of certified EV standards that define particle counts and molecular compositions. The variability of SERS “ hotspots constrains quantitative accuracy despite maintaining high sensitivity. Most systems require skilled operators and advanced instrumentation, which limits their widespread application in POC settings [81,82,83,84,85]. Future success depends on standardized nanofabrication protocols, validated antifouling surface chemistries, miniaturized user-friendly devices, and comprehensive clinical validation that confirms the diagnostic utility beyond analytical performance alone [81,82,84,85].

### 5.3. Micro/Nanofluidics and Single-Vesicle Counting

Microfluidic technologies facilitate integrated processes for separation, purification, and detection of EVs in compact automated systems [88,89,90,91,92,93,94]. Innovative designs allow these devices to attain a sensitivity of 9 EVs/μL with single-vesicle precision [89,91]. Utilizing 300 pL single-cell secretion chambers in conjunction with multicolor TIRF microscopy, EVs were categorized into 15 distinct phenotypic subgroups [91]. Nanoporous silicon nitride membranes facilitate rapid detection of rare EVs in catch-and-display assays [88]. A droplet digital ELISA (ddELISA) operates at a rate of 20 million droplets per minute, demonstrating speed and sensitivity that surpasses traditional assays by a factor of 100 [89]. Silicon nanowire sensors demonstrate a detection limit of 2 × 10^5^ sEVs/mL, effectively isolating, enriching, and quantifying distinct populations of EVs in minimal sample volumes [88,92]. Advanced single-particle tracking methods, including fluorescence, metal nanoparticles, and label-free approaches, facilitate real-time imaging of EVs [88,91,92]. Advancements in clinical translation. Digital microfluidic devices employ automatic biofluid processing and electrochemical readout to assess immunotherapy by detecting PD-L1^+^ EVs [90,91]. Microfluidic extraction techniques effectively prepare platelet-free plasma for investigating multi-omics EVs [89,90,94]. Label-free detection makes integrated lab-on-chip devices feasible for POC applications [88,91,92]. Microfluidic technologies for the isolation of EVs are progressing as they obtain CE markings and FDA approval, leading to advancements in commercial platforms [93,94]. These versatile systems facilitate disease diagnosis, treatment assessments, and pharmacokinetic studies. The primary advantages consist of diminished sample volumes (<100 μL), expedited processing durations (<6 h for 384 samples), potential for automation to minimize operator variability, and compatibility with multiomics profiling [88,89,90,91,92,93,94]. However, substantial translational challenges remain. Scalability is constrained by tradeoffs related to throughput and sensitivity. High-throughput methods trade-off molecular details, whereas high-resolution single-vesicle techniques sacrifice speed. Injection molding and 3D printing are inadequate for replicating the nanoscale characteristics essential for effective EV capture, resulting in challenges related to bulk production that are both difficult and costly. The recovery of EVs is diminished because of the sample pretreatment aimed at reducing clogging. Affinity-based methods select subsets, whereas physical techniques collect lipoproteins and aggregates. Interdevice variability results in significant laboratory discrepancies due to insufficient standardization. Limited microfluidic EV diagnostic assays have received clinical approval, and their regulatory processes are not well defined. The integration of isolation, detection, and analysis with scalable manufacturing and multicenter validation is essential to demonstrate diagnostic efficacy and cost-effectiveness for clinical impact [88,89,90,91,92,93,94].

## 6. Performance Benchmarking, Multiplexing, and Data Analytics

### 6.1. Analytical Metrics, Controls, and Interlaboratory Comparability

**Figure 4:**
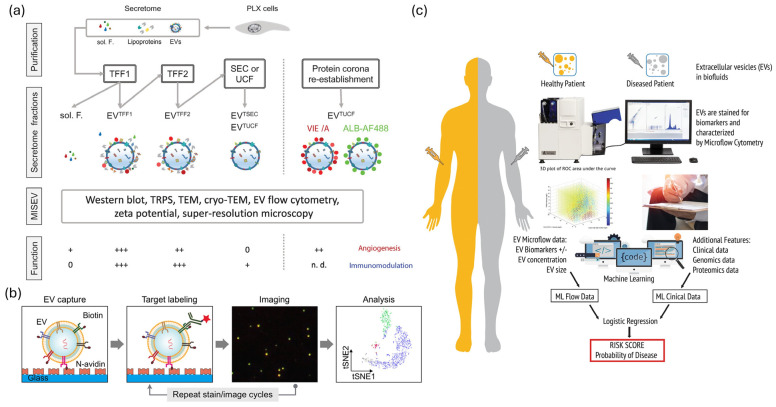
Standardization, multiplexing, and machine-learning strategies in clinical EV biosensing. (**a**) Standardization workflow for EV analysis based on MISEV 2023 and EV-TRACK guidelines. Adapted from [107]. (**b**) Multiplexed single-EV assay combining fluorescence imaging and bead-based detection. Reproduced with permission from [16]. (**c**) Machine-learning pipeline integrating multi-omics EV data for diagnostic prediction. Adapted from [108].

Standardization frameworks are evolving to meet the requirements of clinical EV biosensing [95,96,97,98,99,100]. The MISEV2023 guidelines address EV nomenclature, isolation protocols, characterization requirements, and functional assays [97,98] (Figure 4a). In contrast, EV-TRACK serves as a centralized knowledge base that provides checklists to enhance methodological transparency and facilitate meta-analyses across studies [95,96] (Figure 4a). Studies validating fiber-optic SPR indicate that recombinant extracellular vesicles (rEVs) serve as effective reference materials for biosensor calibration [82,83,84]. Performance indicators such as accuracy, precision, detection limit, linearity, specificity, and repeatability are frequently presented alongside practical characteristics, including analysis time, sample volume, and cost-effectiveness [101,102]. Recent electrochemical platforms utilizing machine learning have demonstrated a clinically relevant diagnostic accuracy of 88.3% and efficiency with an AUC value of 0.883 for early cancer detection [103,104,105]. In contrast, SPR biosensors exhibit exceptional precision, with a coefficient of variation ranging from 3.5% to 9.3% and recovery rates between 101% and 105% in the analysis of clinical samples [82,83,84,85]. Multiplexed biosensor arrays enable simultaneous quantification of inflammatory biomarkers with sensitivity and specificity similar to those of immunoassays [82,84,102]. Commercial platforms are establishing quality control protocols and are undergoing regulatory assessments, with multiple EV-based diagnostics obtaining clinical approval or CE certification [80,93,94,101]. Innovations in biosensor design, including the integration of nanomaterials, microfluidic coupling, and signal amplification, have enhanced the analytical performance of fluorescence, electrochemical, SPR, and magnetic methods, when paired with optimal enrichment procedures [93,104,105]. Substantial gaps in standards persist; less than 40% of published studies on EVs offer adequate methodological details for replication [95,96,97,98]. Compliance with the MISEV2023 guidelines is voluntary and varies significantly among the studies [97,98]. Certified reference materials with defined particle concentrations, size distributions, and molecular compositions are scarce [95,96,97,99]. Furthermore, analytical validation remains a significant issue. The ExoDx Prostate IntelliScore test, now FDA-cleared, is significant for its reliance on data from over 1500 individuals to determine its diagnostic sensitivity, specificity, and predictive value across large patient cohorts [106]. Pre-analytical variables, such as sample collection, processing delays, and storage conditions significantly affect EV profiles; however, these factors are often underreported in the literature [95,96,97,98,99,100]. Ring experiments exhibited variability exceeding two-fold among different laboratories [95,96,97]. To attain clinical-grade repeatability (CV < 10–15%), it is essential to implement standard operating procedures, utilize approved reference materials, maintain consistent reporting formats, and engage in community-wide benchmarking [95,96,97,98,99,100].

### 6.2. Multiplexed and Multiomic Assays

EV multiplexing is an effective method for addressing disease heterogeneity and biological complexity [99,100,101,102]. Plasmon-enhanced systems enable the profiling of various surface proteins at a single-EV resolution, effectively addressing limitations associated with epitope abundance and surface area [81,82,85,102]. Advanced fluorescence methods enabled five-cycle multichannel staining of 15 EV biomarkers, demonstrating that markers independently designate subtypes, rare subpopulations serve as diagnostic indicators, and deep profiling enhances clinical resolution (Figure 4b) [102,109,110]. Western blotting and bead-based multiplexing facilitate the cross-species analysis of EVs from low-abundance samples, employing validated 9-antibody panels on hybrid systems, such as DigiWest (Figure 4b) [85,102]. High-throughput single-EV liquid biopsy devices require minimal sample volume (~90 μL), offer rapid processing times (~6 h), and support scalability (384 samples per run) for the simultaneous detection of miRNAs, mRNAs, and proteins [89,92,94,99,100,101]. The integration of proteomic, transcriptomic (miRNA, mRNA, and lncRNA), lipidomic, and glycomic datasets offers valuable insights into the functions of EVs, their tissue origins, and associated disease processes [99,100,111]. Clinical applications demonstrate significance: Next-generation EV biomarker panels analyze over 4 million CpG methylation sites, 20,000 protein-coding RNAs, and lncRNAs through hybrid capture, 18–200 nt small non-coding RNAs, and 552 cancer-related proteins using Olink platforms, demonstrating superior performance compared to cell-free DNA for early cancer detection [99,100,101]. Spatially resolved multi-omics profiling combines RNAscope and sequential immunofluorescence to delineate RNA targets and proteins within individual cells for the identification of ADC targets Multiple multi-analyte EV tests possess CE marking and are currently undergoing evaluation in the fields of oncology, neurology, and cardiology [80,93,94,101]. Methods for feature selection, multivariate modeling, and integrative data analysis pipelines are advancing to effectively manage high-dimensional multiomics datasets [99,100,103,110]. Multiplexing presents several challenges: signal crosstalk, spectral overlap, and electrode interference diminish sensitivity for low-abundance targets; optimization conflicts arise when one biomarker is optimal, while others are not; and statistical overfitting frequently occurs, with complex panels often failing external validation owing to noise in discovery cohorts [99,100,102,103]. Multiomics integration presents a technological challenge. Data normalization, batch correction, and cross-platform integration remain underdeveloped, whereas EV cargo sorting and functional heterogeneity pose challenges for biological interpretation [99,100,110,111]. Laboratory reproducibility is inadequate, regulatory pathways for multi-analyte panels are intricate (necessitating the validation of components), and financial constraints along with workflow complexity, hinder regular clinical application [80,93,94,99,100,101]. To advance translation, there is a need for standardized marker panels, validated integration algorithms, streamlined high-throughput devices, and comprehensive evidence demonstrating that multimarker signatures enhance diagnostic value beyond traditional clinical measures [99,100,101,103,110].

### 6.3. Computational Analysis and Machine Learning for EV Signatures

Machine learning has revolutionized EV diagnostics by identifying clinically significant patterns in extensive high-dimensional datasets [99,100,103,104]. Machine learning transforms electric-car diagnostics by identifying clinically significant patterns in high-dimensional datasets [99,100,103,104]. Dual-mode EV-circRNA analyzers employing rolling circle amplification and machine learning achieved an accuracy of 88.3% (AUC 0.883) in differentiating gastric cancer patients from healthy donors (Figure 4c), while electrochemical miRNA panels (miR-1246, miR-21, miR-183-5p, miR-142-5p) surpassed single-marker diagnostics for early tumor detection and treatment monitoring, and self-powered electrochemical biosensors integrated with artificial intelligence [74,75,103,104,105]. By employing machine learning to identify patterns in ostensibly random EV datasets, multiomics integration frameworks can elucidate biological and technological variability, thereby enhancing the understanding of systems-level EV biology [99,100,103,110]. Commercial deployment has expanded, and electrochemical sensors powered by AI are practical for oncological diagnosis [101,103,104,105]. Platforms for detecting exosomal miRNAs augmented by machine learning may assist in the diagnosis of neurodegenerative diseases [103]. Computational pipelines for multiomics EV profiling, encompassing methylation, RNA, and protein analyses, reveal biomarker patterns that surpass cell-free DNA in early detection [99,100,101,110]. Mechanistic model predictions enhance clinical and regulatory trust through explainable AI technologies such as SHAP and LIME (Figure 4c). Standards such as containerization and workflow management enhance computational reproducibility, whereas cloud-based platforms facilitate model sharing and validation [99,100,103]. Overfitting frequently occurs in the literature owing to high-dimensional EV datasets and limited sample sizes, resulting in models that capture noise rather than biological signals. External validation is infrequent, cohort-specific biases result in model failures when evaluated on independent populations, feature selection biases exaggerate performance estimates prior to model training, and there is inadequate documentation of data preprocessing decisions [99,100,103]. The clinical utility of enhanced patient outcomes in prospective randomized trials beyond analytical performance metrics remains unsubstantiated [99,100,103]. Clinical adoption necessitates multicenter validation with varied cohorts, transparent models, data-sharing protocols, criteria for computational reproducibility, clarification of the regulatory environment, and definitive evidence that EV-ML diagnostics enhance patient care [97,98,99,100,103].

## 7. Clinical Translation and Future Outlook

### 7.1. Disease Applications: Oncology, Neurology, Cardio-Metabolic, Infectious Diseases

EVs function as reflectors of intercellular pathological changes and signaling by carrying various biomolecules, such as proteins, nucleic acids, and lipids. This property enables EVs to serve as prognostic and diagnostic biomarkers in a wide range of clinical fields, including neurological diseases, cancer, infectious diseases, and cardiometabolic disorders. In oncology, EVs regulate interactions between immune and tumor cells within the tumor microenvironment, and specific protein signatures or miRNAs contained in tumor-derived EVs have been reported to be useful for predicting metastatic potential and therapeutic responsiveness. For example, PD-L1 expression on large EVs has been proposed as an indicator of immune-therapy response, even in patients with PD-L1-negative tissues. Specifically, the detection of glypican-1-positive EVs has demonstrated an area under the curve (AUC) of 1.0 for identifying early-stage pancreatic cancer, significantly outperforming conventional serum markers [112]. Furthermore, quantitative monitoring of exosomal PD-L1 has shown that a significant increase in circulating PD-L1^+^ EVs can predict immunotherapy resistance in melanoma patients, allowing for more precise therapeutic adjustments. Circulating EV-based liquid biopsy is emerging as a non-invasive diagnostic method that can replace conventional tissue biopsies. Circulating EV biomarkers have shown promising performance in cancer liquid biopsy, with pooled sensitivity and specificity of ~84% and ~86% in prostate cancer diagnosis, and multi-marker EV miRNA/protein panels achieving AUC values > 0.9 for treatment response classification [113]. In neurological diseases such as Alzheimer’s and Parkinson’s disease, the ability of EVs to cross the blood–brain barrier (BBB) has attracted increasing attention. Proteins such as tau, Aβ, and α-synuclein within EVs can be utilized for the staging and early diagnosis of neurodegenerative diseases, and drug delivery through EVs is also being developed as a therapeutic strategy targeting the brain. Quantitative analysis indicates that p-Tau181 and Aβ42 levels within neuron-derived EVs can predict the onset of Alzheimer’s disease with over 90% accuracy up to ten years before clinical symptoms manifest [114]. This high predictive value highlights the potential of EV-based assays as a non-invasive alternative to cerebrospinal fluid sampling for early neurodegenerative staging. EVs have been proposed as biomarkers of metabolic stress, including inflammation, cardiomyocyte injury, and insulin resistance in cardiovascular and metabolic diseases. Reports have shown that Adipocyte-derived EVs are useful for monitoring glucose metabolism abnormalities, whereas circulating endothelial-derived EVs are valuable for predicting myocardial infarction. In cardiovascular contexts, muscle-specific miRNAs such as miR-1 and miR-133a are significantly enriched in circulating EVs within a 2–4-h window following myocardial injury, enabling much earlier detection than traditional cardiac troponin assays [115]. Similarly, adipocyte-derived EVs have been quantitatively mapped to monitor insulin resistance and glucose metabolism abnormalities with high sensitivity. In the field of infectious diseases, EVs reflect host responses following pathogen infection, and it has been elucidated that viral RNA or protein transmission via EVs contributes to immune evasion during viral infection. EV-based sensing technologies have been developed into practical tools for the rapid detection and disease monitoring of such infectious agents. Ultimately, EVs have strengthened their clinical applicability as key biosources in precision medicine, including therapeutic response monitoring, early diagnosis, and prognostic evaluation [1,7,116,117,118,119,120]. For instance, CRISPR-integrated biosensing platforms have achieved ultra-sensitive detection of SARS-CoV-2 spike proteins with a limit of detection as low as 0.1 fg/mL [121]. Such high sensitivity facilitates the identification of viral components even in patients with extremely low viral loads, significantly improving the early diagnostic capabilities for infectious agents.

### 7.2. Standardization and Regulatory Pathways (MISEV, EV-TRACK, IVD/ISO)

Standardization and regulatory compliance in the isolation, quantification, and analysis processes are essential for the clinical translation of EV research. Since the physicochemical properties of EVs vary depending on the origin of the cells, isolation methods, and storage conditions, global guidelines have been established to ensure the reproducibility and comparability of results.

The ISEV proposed the “MISEV” guidelines, which define the minimum requirements for EV isolation, identification, and characterization. Accordingly, the identification of EV surface markers (CD9, CD63, CD81, etc.); quantification of proteins, RNA, and lipids; and transparent reporting of experimental conditions have been emphasized. Additionally, the EV-TRACK database serves as a platform to enhance the reproducibility and reliability of EV studies by allowing researchers to register their experimental information publicly. Data sharing is an important method of ensuring reliability during the development and regulatory approval of clinical EV-based diagnostic devices. From a regulatory perspective, EV-based diagnostic devices must comply with the in vitro diagnostic (IVD) and ISO 13485 [122] quality management system standards, and the establishment of GMP environments for clinical-grade manufacturing is required. The U.S. FDA and European EMA are gradually establishing approval guidelines for new technologies, such as EV-based liquid biopsy and drug delivery systems. To further promote the clinical application of EV research, it is necessary to standardize isolation technologies (e.g., size-exclusion chromatography and ultrafiltration), introduce large-scale automated systems, and establish internationally certified reference materials for EVs. These regulatory and standardization frameworks serve as a foundation for the dissemination of EV biosensing technologies in clinical settings [9,96,123].

### 7.3. Manufacturing, Scale-Up, Point-of-Care Integration, and Future Directions

The key to the commercialization and clinical success of EV biosensors lies in their large-scale manufacturing, automated and precise quality control, POC applications, and future innovation strategies. Recently, large-scale production based on bioreactors and the establishment of intelligent manufacturing lines (including GMP implementation and batch-to-batch comparability) have achieved consistency and reproducibility that are suitable for clinical use and diagnostic device integration. Automated production and isolation platforms, cost-effective microfluidic fractionation, and new technologies for targeted biomarker collection have significantly improved production efficiency and have been incorporated into the commercial manufacturing lines of EV biosensor companies. POC diagnostic platforms also maximize patient accessibility and efficiency through rapid in-hospital diagnosis, home testing, and decentralized regional examinations. In future, synthetic EV platforms, AI-based real-time quality control, remote monitoring, and globally automated standardized distributed manufacturing will be introduced. Unresolved issues, such as targeted design, long-term stability, and distributed production networks, are expected to be addressed simultaneously. The core elements of commercial success include the standardization of GMP, QC, and distributed production, as well as the establishment of innovative systems consistent with global data integration and regulatory trends. These convergent advances will contribute to the establishment of EV-based biosensing as a key axis for next-generation precision diagnostics and personalized medicine [9,96,123]. Despite the rapid advancement of EV biosensing, a significant gap remains between laboratory innovation and commercial implementation. Bulk-based detection techniques, while high-throughput, provide only ensemble-averaged data that mask the intrinsic heterogeneity of EV populations [124]. This ‘average trap’ often leads to false negatives in early-stage diagnostics where disease-specific EVs are present in low concentrations. Conversely, single-molecule and single-EV technologies offer the resolution necessary to resolve these subpopulations; however, they are currently hindered by the requirement for expensive instrumentation, highly skilled operators, and low sample throughput [125]. These logistical and financial barriers represent a major bottleneck for the translation of single-EV platforms from the bench to the clinical market, necessitating the development of automated, cost-effective hybrid systems.

## 8. Conclusions

EV biosensing has emerged as a dynamic and rapidly advancing frontier in biomedical diagnostics, integrating the biological complexity of EVs with the precision and scalability of modern analytical technologies. By integrating molecular recognition elements, advanced signal transduction mechanisms, and antifouling surface chemistry, EV biosensors enable highly sensitive, selective, and multiplexed detection at the single-vesicle level. These technological advances have significantly expanded the diagnostic utility of EVs, providing novel approaches for non-invasive disease monitoring and therapeutic evaluation in oncology, neurology, infectious diseases, and cardiometabolic disorders. However, despite these achievements, several challenges remain before EV biosensing is fully integrated into clinical practice. The intrinsic heterogeneity of EVs, variability in isolation and quantification methods, and the lack of standardized reference materials continue to hinder cross-laboratory reproducibility and clinical validation. To overcome these obstacles, global initiatives, such as MISEV and EV-TRACK, have laid the essential foundations for methodological transparency and reproducibility, while regulatory alignment with IVD and ISO standards is gradually facilitating clinical translation. Furthermore, the establishment of GMP-compliant manufacturing environments and automated production platforms is indispensable for ensuring clinical-grade quality and scalability. Looking ahead, the convergence of EV biosensing with artificial intelligence, microfluidic automation, and next-generation nanomaterials is expected to enable real-time, high-throughput, and ultrasensitive analyses compatible with POC settings. Future developments are shaped by synthetic EV platforms, adaptive sensor architectures, and distributed manufacturing networks supported by integrated data systems. These innovations will accelerate the transition of EV biosensing from experimental research to standardized clinical diagnostics, ultimately contributing to the realization of predictive, preventive, and personalized precision medicine. EV-based biosensing is a transformative paradigm that bridges the fields of molecular biology, materials science, and clinical engineering. Through the continuous refinement of analytical methodologies and harmonization of global standards, EV biosensing has evolved from a promising research tool to a clinically indispensable diagnostic modality, ushering in a new era of individualized healthcare and real-time disease monitoring.

## Figures and Tables

**Table 2:** Comparison of Single EV Biosensing Strategies.

Category	Method/Technology	Key Advantage	Performance/Detection Limit (LOD, particles/mL)	Application	Sample Type	Ref.
Recognition Elements	Dual tetraspanin antibody (CD81/CD63)	Cotargeting improves sensitivity and overcomes EV heterogeneity	~1 × 10^4^–1 × 10^7^ particles/mL	Plasma EV detection; size-bias compensation	Plasma; cell culture supernatant	[66]
	Antibody-aptamer complex (AAC)	High specificity via dual recognition; chemical stability	~1 × 10^3^–1 × 10^6^ particles/mL	Selective isolation of EV subtypes	Cancer cell–derived EVs	[67]
	Aptamer (SELEX-based)	Small size; facile modification; low-cost synthesis	~4 × 10^3^ particles/mL (CD63 aptamer sensor)	PD-L1^+^ EV isolation; immune profiling	Cell culture supernatant	[68]
Transduction & Amplification	EQCM-D with 2D gold nanoarray	Multimodal detection (mass/dissipation); enhanced sensitivity	~1 × 10^5^–1 × 10^6^ particles/mL	Single EV detection in plasma	Plasma; biofluids	[69]
	SERS (label-free)	10^9^–10^11^ × signal amplification; multiplexing capability	~1 × 10^3^–1 × 10^5^ particles/mL	Cancer-derived sEV profiling	Serum; plasma; cell culture medium	[70]
	CRISPR-Cas12a cascade	Enzymatic trans-cleavage enables ultrahigh sensitivity	~1 × 10^2^–1 × 10^3^ particles/mL (reported EV-associated nucleic acid assays)	Liquid biopsy; EV-associated RNA detection	Plasma; biofluids	[71]
	DNA hydrogel-hosted CRISPR	Programmable fluorescence/colorimetric outputs	~1 × 10^3^–1 × 10^4^ particles/mL	POC diagnostics	Buffer; simulated clinical samples	[69]
	3D-AFM nanomechanical mapping	Single-EV nanomechanical characterization	Detection at single-EV level (~10^4^ particles/mL equivalent)	Cancer metastasis mechanobiology	Plasma; liquid samples	[72]
Surface Chemistry & Antifouling	Porous nanocomposite coating	Long-term antifouling; sensitivity enhancement	~1 × 10^4^–1 × 10^6^ particles/mL	Fouling-resistant EV biosensors	Complex biofluids	[5]
	SAM-based coating (Si-MEG-OH)	Reduced nonspecific adsorption; polymer-brush behavior	~1 × 10^5^–1 × 10^6^ particles/mL	Electrochemical/SPR EV sensors	Plasma	[73]
	Peptide self-assembled layer	Improved signal stability and reproducibility	~1 × 10^4^–1 × 10^6^ particles/mL	Electrochemical EV biosensing	Cell culture supernatant; plasma	[5]
	Printed antifouling electrode	Scalable fabrication; disposable platforms	~1 × 10^5^ particles/mL	POC EV sensor fabrication	Blood; clinical fluids	[5]
	MIP-based ratiometric biosensor	Internal reference corrects matrix effects	~1 × 10^4^–1 × 10^5^ particles/mL (particle-equivalent)	Virus/EV-associated protein detection	Serum	[5]
	Self-powered enzymatic biofuel cell sensor	No external power; stable baseline	~1 × 10^5^ particles/mL	Point-of-care EV detection	Plasma; serum	[5]

## Data Availability

No new data was created for this study. All data analyzed in this review are available in the publications cited in the references section.

## Abbreviations

The following abbreviations are used in this manuscript:

AAC	Antibody–aptamer combination/antibody–aptamer complex
ANSWER	Acoustofluidic Nanosorter by Wave-Pillar Resonance
BAWs	bulk acoustic waves
BBB	blood–brain barrier
CRISPR	clustered regularly interspaced short palindromic repeats
ddELISA	droplet digital ELISA
DEP	Dielectrophoresis
dsDNA	double-stranded DNA
DTT	dithiothreitol
ESCRT	Endosomal sorting complex required for transport
EVOD	EVs On Demand
EVs	extracellular vesicles
FLOAT	floating acoustic trapping
ISEV	International Society for Extracellular Vesicles
IVD	in vitro diagnostic
iDEP	Insulator-based dielectrophoresis
lncRNAs	long noncoding RNAs
MBVs	matrix-bound nanovesicles
miRNAs	microRNAs
MISEV	minimal information for studies of Extracellular Vesicles
MIPs	molecularly imprinted polymers
mRNAs	messenger RNAs
mtDNA	mitochondrial DNA
MVBs	multivesicular bodies
MSCs	mesenchymal stem cells
PEG	polyethylene glycol
POC	point-of-care
PS	phosphatidylserine
rEVs	recombinant extracellular vesicles
SAWs	surface acoustic waves
SEC	Size-exclusion chromatography
SELEX	systematic development of ligands using exponential enrichment
sEV	small extracellular vesicle
SPR	surface plasmon resonance
ssDNA	single-stranded DNA
TEI	Total Exosome Isolation
UC	Ultracentrifugation
